# Laboratory study on "intracranial hypotension" created by pumping the chamber of a hydrocephalus shunt

**DOI:** 10.1186/1743-8454-4-2

**Published:** 2007-03-26

**Authors:** Adam Bromby, Zofia Czosnyka, David Allin, Hugh K Richards, John D Pickard, Marek Czosnyka

**Affiliations:** 1 Academic Neurosurgical Unit, Addenbrooke's Hospital, Cambridge, CB2 0QQ, UK

## Abstract

**Background:**

It has been reported that pumping a shunt *in situ *may precipitate a proximal occlusion, and/or lead to ventricular over-drainage, particularly in the context of small ventricles. In the laboratory we measured the effect of pumping the pre-chamber of hydrocephalus shunts on intracranial hypotension.

**Materials and methods:**

A simple physical model of the CSF space in a hydrocephalic patient was constructed with appropriate compliance, CSF production and circulation. This was used to test eleven different hydrocephalus shunts. The lowest pressure obtained, the number of pumps needed to reach this pressure, and the maximum pressure change with a single pump, were recorded.

**Results:**

All models were able to produce negative pressures ranging from -11.5 mmHg (Orbis-Sigma valve) to -233.1 mmHg (Sinu-Shunt). The number of pumps required reaching these levels ranged from 21 (PS Medical LP Reservoir) to 315 (Codman Hakim-Programmable). The maximum pressure change per pump ranged from 0.39 mmHg (Orbis-Sigma valve) to 23.1 (PS Medical LP Reservoir).

**Conclusion:**

Patients, carers and professionals should be warned that 'pumping' a shunt's pre-chamber may cause a large change in intracranial pressure and predispose the patient to ventricular catheter obstruction or other complications.

## Background

It has been reported previously that hydrocephalus shunts may cause over-drainage, in particular, during changes in posture. Kajimoto *et al *[1] postulated that this over-drainage was due to increased hydrostatic pressure in the ventriculoperitoneal shunt system. This increases the differential pressure acting across a shunt of relatively low hydrodynamic resistance [2] and may provoke excessive drainage, leading to intracranial hypotension.

The pumping of a shunt's pre-chamber has been used to test shunt patency [3]. However, the specificity and sensitivity of such testing were assessed as not satisfactory. Historically, some patients and their families were encouraged to pump the shunts periodically to avoid blockage of the valve or to relieve headaches. However, such a maneuver may possibly lead to over-drainage.

Low intracranial pressure may result in headache, nausea and vomiting, diplopia, lethargy, paresis of upwards gaze and strabismus, dizziness and hearing disturbances. These symptoms mainly occur when the patient is upright and active [4]. The orthostatic headache is thought to be the result of a downward displacement of the brain. When a person is upright, the brain is kept afloat by the buoyant action of the CSF in conjunction with the anchoring effects of the vascular structures in the cranium; if the buoyant action of the CSF is decreased i.e. when the CSF volume is decreased, the burden on the vascular structures increases resulting in traction and distortion [5]. Diencephalic compression of the brain due to downward dislocation has also been reported to decrease consciousness [6,7]. As these vascular structures in the cranium are pain-sensitive [8], orthostatic headache occurs. In terminal conditions, CSF hypovolaemia may result in subdural haemorrhage due to the tearing of the bridging veins as the brain pulls away from the dura [9,10]. However, there is no clinical report of this happening following shunt pumping.

CSF drainage through some shunt systems, may be accelerated by 'pumping' the shunt pre-chamber. So far, 'pumping' has been evaluated quantitatively with results reported in the form of various conference presentations [11,12], but not in peer-reviewed journals. We have built a physical model of CSF circulation and compensation, 'shunted' it and investigated eleven shunts in the laboratory, to determine to what extent CSF pressure may be reduced by repetitive pumping.

## Materials and methods

### Shunts

We tested eleven types of shunt, either new or previously evaluated in the UK Shunt Laboratory (none were explanted from patients). Types, manufacturers and other details are given in Table 1.

**Table 1:** Name, manufacturer, sub-type, performance levels, and catalogue numbers of tested shunts.

Name	Manufacturer	Sub-types tested	Performance level	Catalogue Number
Hakim-Precision Valve	Codman	Micro Valve and standard	Medium	82–3028 82–3013
Hakim Programmable	Codman		3 cmH_2_O and 20 cmH_2_O	82–3110
Hakim Programmable with Siphon-Guard	Codman		3 cm H_2_O and 20 cmH_2_O	82–3162
Delta	Medtronic, USA		Performance 1	42822
Strata	Medtronic, USA	Small and regular	Performance 0.5 and 2.5	42866
LP reservoir	Medtronic, USA	30 mm		44515
Flow Control	Medtronic, USA	Contoured, standard, burr-hole	Medium pressure	42324 42104 42534
Diamond	Phoenix, USA			Unknown, (only one type available)
Pedi Gav	Miethke, Germany	With reservoir	9/24 cmH_2_O	FV 306T
Orbis-Sigma	Integra Neuroscience Implants, France			811201
Sinu-Shunt	CSFdynamics, Denmark			Unknown, (only one type available)

All shunts were differential pressure valves with the exception of the Orbis-Sigma valve and the Diamond valve, which work by a principle of stabilizing flow over wide range of differential pressures.

The Codman Programmable Valve (with Siphon-Guard), the Medtronic Delta and Strata Valves all had siphon-preventing devices.

### Testing model

**Figure 1:**
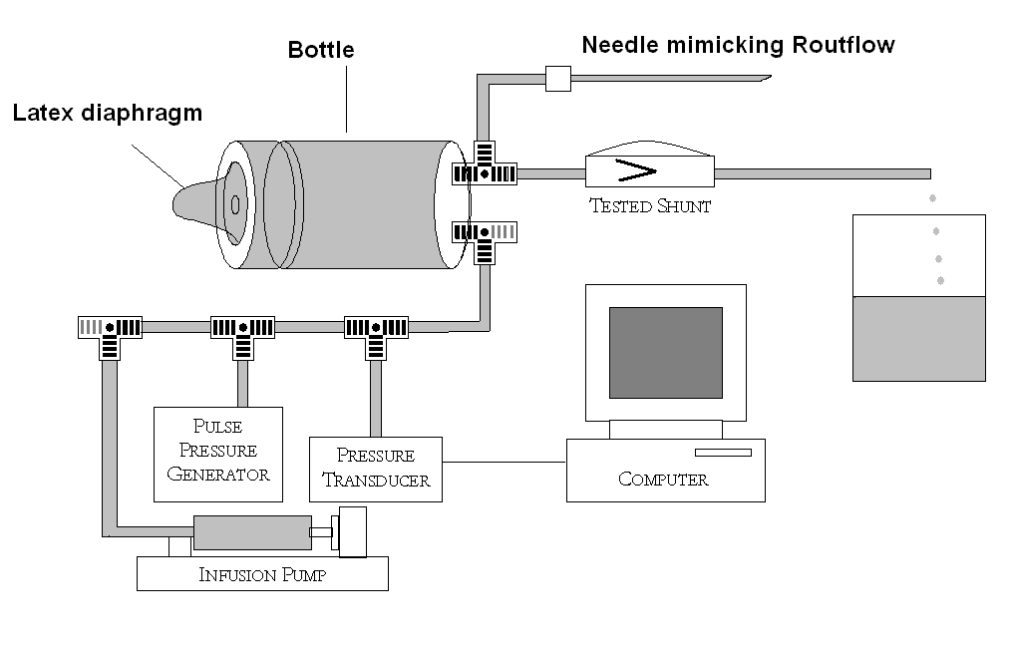
The laboratory rig used to test the pumping actions of hydrocephalus shunts including model of CSF compensation.

To model the cerebrospinal fluid (CSF) space, a wide-necked feeding bottle (270 ml, Boots, UK) was filled with de-aerated de-ionised water to mimic CSF (Fig. 1). A latex membrane (0.14 mm thick, diameter 4 cm) was placed over the top of the bottle and under the cap. A compliant system was created by making a 2 cm diameter hole in the cap. The fluid forced the membrane through the hole and the size of the hole determined the magnitude of the compliance. The model also included a resistance to CSF reabsorption in the form of a lumbar puncture outflow needle, giving a resistance to CSF flow of 7.4 mmHg ml^-1^min^-1^, within the 6–10 mmHg range found in humans [13,14]. To mimic CSF production, fluid was infused at a constant rate of 0.3 ml min^-1^. The shunt to be tested was attached to the model using 10 cm of low resistance tubing, mimicking a ventricular catheter. An outflow of the same tubing (80 cm long and 1.2 mm ID) mimicked the peritoneal catheter.

**Figure 2:**
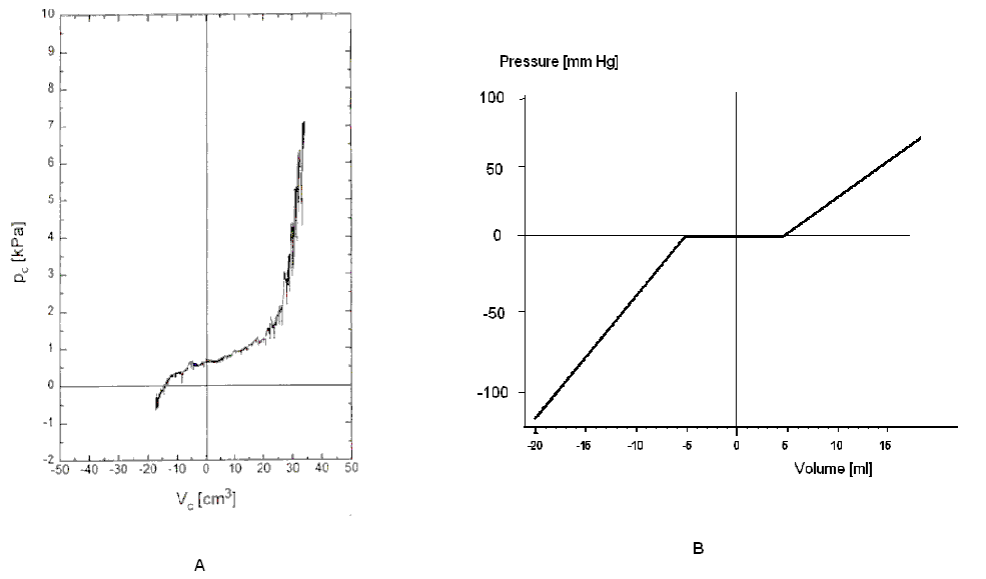
Cerebrospinal pressure-volume curves. a. Pressure-volume curve plotted using clinical test- with permission from author [13]. b. Pressure-volume curve of the model used for testing *in vitro*. Three-part approximation of the compliance within the CSF compartments. The compliance over the first part is 0.17 ml mmHg^-1^, over the plateau 5.53 ml mmHg^-1^, over the final part 0.31 ml mmHg^-1^. This is computer graph was generated to interpolate measured values.

The *in vivo *CSF pressure-volume curve has a symmetrical exponential shape according to Friden [13] (Fig. 2a). The pressure-volume curve of the model had three approximately linear zones (Fig. 2b) characterized by a compliance of 0.17 ml mmHg^-1 ^within the range of negative pressures, 5.53 ml mmHg^-1 ^during the plateau and 0.34 ml mmHg^-1 ^within the range of positive pressures. The compliance was therefore pressure-independent for pressures below -5 mm Hg, between -5 mmHg and 5 mm Hg, and above 5 mm Hg. The compliance of the model was selected to be lower than the values previously reported in the literature (from 1.83 ml mmHg^-1 ^in normal children to 0.97 ml mmHg^-1 ^in children with acute hydrocephalus [15]). The model was designed to mimic the 'worst case', i.e. a system with low pressure-volume compensatory reserve. Greater compliance (0.91 ml mm Hg^-1 ^at the negative pressure range) was also tested, with two valves producing the minimal (Orbis-Sigma) and maximal (Sinu-Shunt) intracranial hypotension during pumping, to check whether compliance may affect the depth of intracranial hypotension.

Pressure was recorded using a Gaeltec Luer Lock transducer with an accuracy of pressure measurement better than +/- 5 mm Hg over the range -250 to 250 mm Hg. A pressure waveform calibrator was used to simulate the ICP pulse pressure with amplitude of 1 mmHg and a rate of 90 beats per minute.

### Protocol

**Figure 3:**
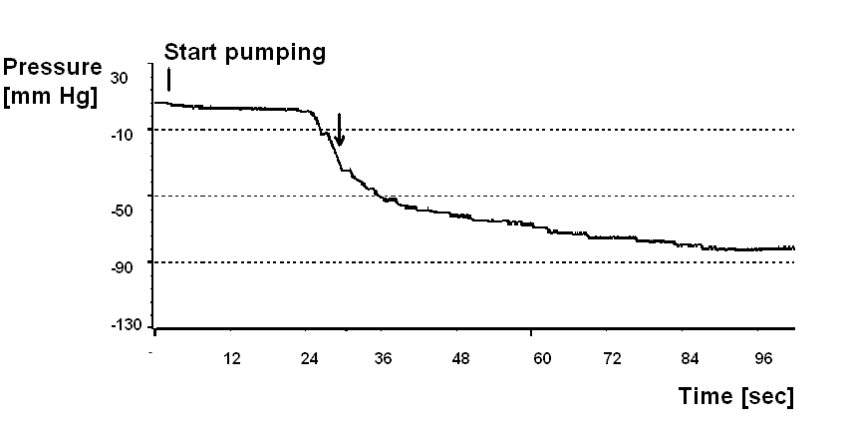
Single test of the PS Medical Lumboperitoneal Reservoir using the model of human CSF space. Pumping started at the vertical bar. Pressure decreased slowly at first on the plateau (Fig. 2b- high-compliance section of the pressure-volume curve) and then started to accelerate (on the steep, low-compliance section of pressure-volume curve), and then it reached the asymptote. Arrow indicates the region where pressure changes are greatest per one pump and these values are taken for comparison between valves (see Fig 6).

For all valves, the negative pressure in the model mimicking the intracranial pressure (ICP) at which an asymptote occurs, was determined by pumping the shunt reservoir continuously, at a constant rate of 1s^-1^, until the asymptote was reached (Fig. 3). The PS Medical Lumboperitoneal shunt had the largest reservoir and could only be pumped at a rate of 1 stroke per 3s, due to the longer refill time. The negative pressure achieved was measured from the data recorded in BioSAn for Win95 [16].

The number of pumps taken to reach the asymptote was measured from the 'switching point' where the model changed from the high compliance of the plateau of the pressure-volume curve to that of the first slope to the point where an asymptote was reached (Fig. 3).

The maximum pressure change in a single pump (ΔP_max_/pump) was measured at the point of the maximum pressure change over time on the recorded curve, i.e. where the curve like in Fig. 3 had minimal first derivative. One sample of each kind of valve was tested. The test was repeated five times for each valve.

### Statistics

The One-Way Analysis of Variance (ANOVA, Tukey test) was used where the normality test was passed. Where this was not passed, the Kruskal-Wallis One-Way ANOVA on Ranks (Dunn's Method) was used.

## Results

**Figure 4:**
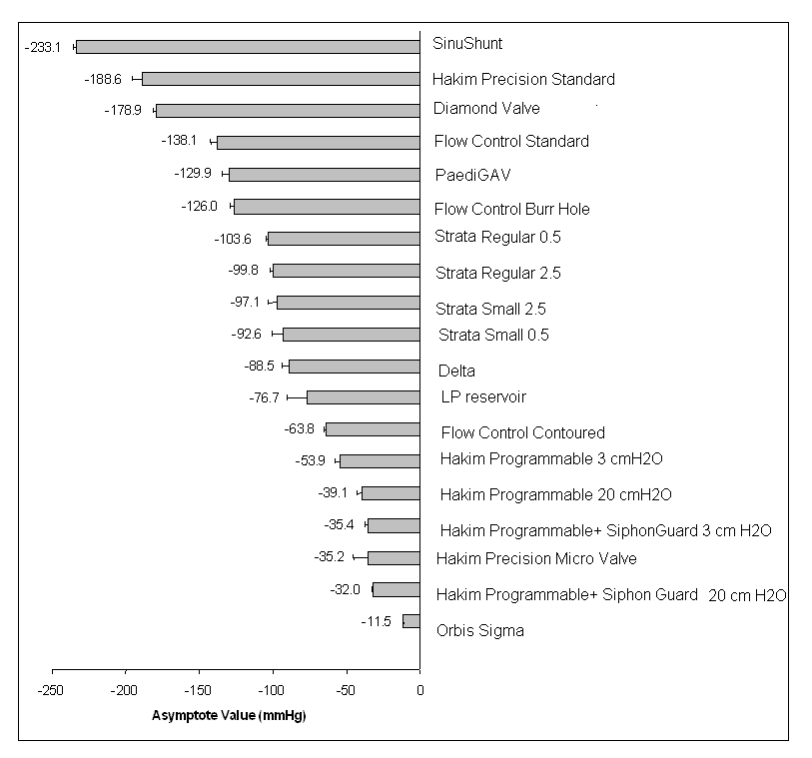
Bar chart showing the pressures for the various valves at which the asymptote was achieved by continuously pumping the reservoirs.

The lowest pressures obtained at the asymptotes following continuous pumping of the valve reservoirs revealed a range of differences between the shunts (Fig. 4). All shunts were capable of creating negative pressures. The shunt able to achieve the lowest negative pressure was the Sinu-Shunt (-233.1 mmHg). The shunt that achieved the least negative pressure was the Orbis-Sigma valve (-11.5 mmHg). These values are significantly different (*P *< 0.05 One-way ANOVA on Ranks). The Codman Non-Programmable Valve produced a significantly lower pressure when using the large pumping chamber (regular Codman-Hakim valve) compared to a smaller pre-chamber (pediatric micro valve) (*P *< 0.001 One-way ANOVA and Tukey's test). There was no difference in performance between configurations of the Strata valve or between the different flow control models.

**Figure 5:**
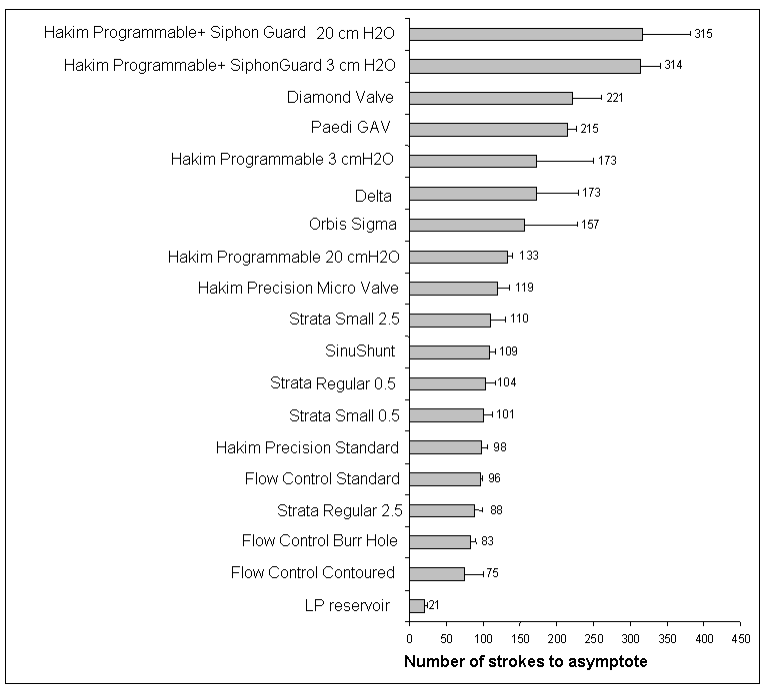
Bar chart showing the number of pumps required for each valve to reach the asymptote.

As it is not clear how the width of the plateau phase of compliance in the model compares with that in human CSF compartments (within the range of negative pressures), the speed at which the asymptotes are reached was assessed from the time of the 'switching point' between high and low compliances (Fig. 5). The fastest performing shunt was the PS Medical Lumboperitoneal valve which required 21 ± 4 pumps to reach the asymptotic negative pressure from the switching point which was significantly (*P *< 0.05 One-way ANOVA on ranks) less than the Codman Programmable Valves at the 2 cmH_2_O and 0.5 cmH_2_O opening pressures which required 315 ± 66 and 314 ± 28 pumps, respectively. The Strata valves showed no difference between models, nor did the Flow Control valves. The Codman valves with or without Siphon-Guards did not perform differently in terms of the number of pumps to reach the asymptote at the high or low pressure settings, but those with the Siphon-Guard did require significantly more pumps to reach the asymptote than their counterparts without Siphon-Guards (*P *< 0.005 at the 2 cm H_2_O settings and *P *< 0.02 at 0.3 cm the H_2_O settings).

**Figure 6:**
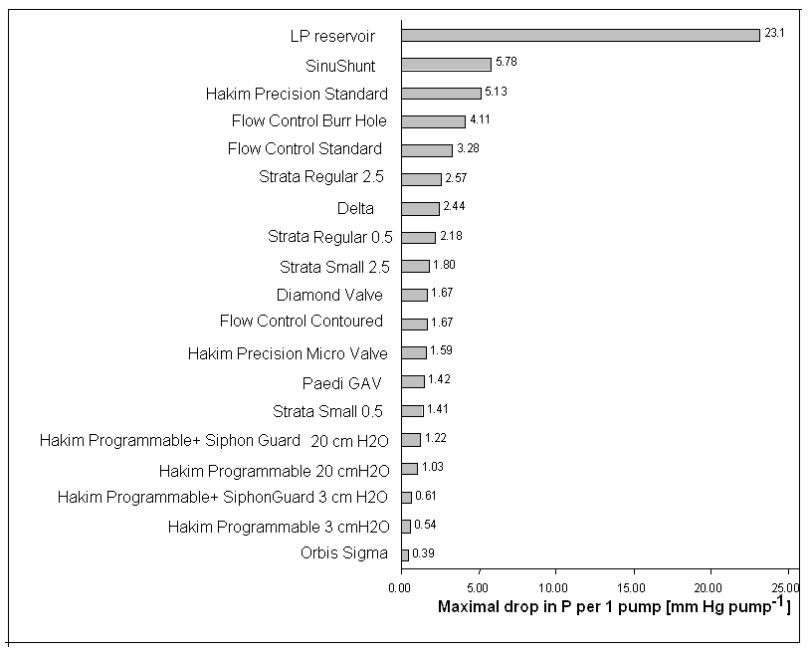
Bar chart showing the maximum pressure reduction achievable with a single pump on the valve's pumping chamber.

The maximum pressure change in a single pump was also measured for each valve (Fig. 6). The valve able to create the largest pressure change was the PS Medical Lumboperitoneal valve. This valve was able to reduce pressure by 23.1 mmHg in a single pump, compared to the Orbis-Sigma valve, which reduced pressure by a maximum of 0.39 mmHg pump^-1^.

Two models with a higher compliance were built. The Orbis-Sigma valve and the Sinu-Shunt were retested using these models. The asymptotes achieved were not significantly different on each model with the same shunt. The time to reach the asymptote was not significantly different when testing the Orbis-Sigma valve but was significantly longer in the Sinu-Shunt (*P *< 0.001 One-way ANOVA Tukey test).

## Discussion

It can always be disputed whether *in-vitro *testing is clinically relevant. We used a model that increased the volume of CSF in a system that had a total volume of 270 ml. This volume is not uncommon in hydrocephalus with gross ventricular dilatation. The model has a relatively low compliance and the width of the horizontal section of pressure-volume curve was chosen somewhat arbitrarily. There is little data regarding this width, although some reports give values around 5 ml [13]. Therefore, we think that a comparison of valves in terms 'how many pumps are needed to reach steeper section of P/V curve' or 'how many pumps are safe' may be misleading. The most important message, repeated after other studies [11,12], is that pumping the chamber of any valve has the potential to reduce proximal CSF pressure significantly, to the extent that clinically relevant over-drainage would be possible.

Patients and families should be advised against pumping, particularly when the ventricles are small. It is possible for a physician to test the valve using a single compression: however such tests are unreliable [3,11,12]. The majority of shunts were able to produce an intracranial pressure of less than -30 to -35 cmH_2_O (-22 to -26 mmHg). This falls within the range that can produce orthostatic headaches [17]. It is at this pressure that the vasodilatation compensation for CSF depletion is no longer sufficient, and thus the symptoms of CSF hypovolaemia become apparent [18].

There is a range of differences between the shunts, even between different models from the same manufacturer. In terms of the recorded negative pressures, the Orbis-Sigma was the best performer, and the Sinu-Shunt the worst. The number of strokes required to reach the asymptotes showed that the LP Shunt was the fastest, probably due to its large pumping reservoir. With Codman Programmable valves, the use of the Siphon-Guard, programmed to a higher performance level, increased the number of strokes required to reach the asymptote but did not affect the position of the asymptote.

Testing the two valves on the higher compliance models, demonstrated the validity of the model in terms of modeling the CSF space and compliance in the hydrocephalic patient. This was shown in both the worst-case scenario (low compliance) and in other hydrocephalic situations since the asymptotes remained unchanged. In patients with a higher compliance than the model, it may be expected that they reach the same asymptote but require a longer period of pumping to get there.

Flow control valves (Orbis-Sigma and Diamond Valve) minimize the degree of intracranial hypotension during pumping. The presence or absence of a siphon-control device has no effect on intracranial hypotension caused by pumping.

## Conclusion

Pumping of shunt pre-chambers may cause gross intracranial hypotension in a relatively short time. The number of pumps and time needed for producing possibly detrimentally low levels of ICP depends on the shunt type.

## Competing interests

The UK shunt laboratory had, in the past, research and development agreements with the manufacturers: Codman, Medtronic, Phoenix, Integra, Aesculap (marketing Miethke shunts) and CSFdynamics. This research was not financed by any of these grants.

## Authors' contributions

AB, ZC, HR, and MZ actively participated in planning, design, conducting experiments, analyzing the results and preparing final submission, making them eligible for co-authorship. JDP and DA had important conceptual input into designing the project and preparing the manuscript. All authors have read and approved the final version of the manuscript.
